# Influence of the elastic modulus of a conveyor belt on the power allocation of multi-drive conveyors

**DOI:** 10.1371/journal.pone.0235768

**Published:** 2020-07-07

**Authors:** Yanping Yao, Bisheng Zhang

**Affiliations:** School of Mechanical Engineering, Taiyuan University of Science & Technology, Taiyuan, China; China University of Mining and Technology, CHINA

## Abstract

The implementation of multiple drives for belt conveyors can solve the problems associated with motor overpower and the excessive tension of conveyor belts powered by a single drive. However, multiple drives can suffer from uneven driving power allocation. Among various factors, the selection of the type of conveyor belt particularly affects the power allocation. The current study aims to investigate the influence of the elastic modulus of a conveyor belt on the power allocation of multi-drive conveyors. Based on the Kelvin-Voigt viscoelastic model, a discrete model of an entire machine is established. Kelvin-Voigt software is used to simulate the working conditions of conveyor belts with different elastic moduli under full loads. The driving forces of individual rollers are obtained and then compared. Compared to other types of belts, a steel wire core conveyor belt, whose elastic modulus is relatively high, effectively improves the stability of the conveyor belt under a full load after start-up to achieve a reasonable power allocation. The results of this study provide a foundation for conveyor belt selection for multi-drive conveyors.

## Introduction

Belt conveyors have been extensively applied to bulk material delivery applications in various industries, such as the metallurgy and mining industries, the coal chemistry industry and the food processing industry. In modern industrial enterprises, bulk transportation accounts for approximately 30% of production costs [1]. In response to policies related to energy conservation and consumption reduction, belt conveyors are being developed to accommodate heavy loads, long distances and high speed [2–4].

As the driving force produced by the traditional single-roller belt conveyor cannot satisfy the requirement for long-distance driving, scholars have proposed the multi-roller driving method [5–7]. However, this method tends to cause uneven power allocation among the rollers [8, 9], which results in either overloaded or underloaded individual electric motors, thereby increasing production costs and wasting resources [10].

Numerous factors can affect the power allocation of multi-drive conveyors, such as the topography of the system, drive spacing, elastic modulus of the conveyor belt, wrap angle of the drive pulleys (the central angle corresponded by the arc length of the roller that is wrapped by the conveyor belt), friction coefficient between the conveyor belt and driving roller, and mechanical performance of the driving device [11]. Among these factors, scholars have focused on the start-up time [12], start-up acceleration curve [12–14], and tensioner performance [15–18] while ignoring the performance of the conveyor belt.

A conveyor belt is typically made of a viscoelastic material, which extends elastically under tension. While running in different segments (except for the driving roller segment), the belt has to overcome various resistances, causing its tension to increase continuously. Correspondingly, the belt gradually extends, its speed gradually increases, and its tension and extension at the joining end of the driving roller increase. Because the peripheral velocity of the roller is constant, the belt elongates and slides along the opposing direction of the roller rotation, which leads to the creep of the conveyor belt on the roller [19]. This condition in turn affects the transfer of the peripheral force of the driving roller, i.e., power allocation. To improve the performance of the conveyor, the extension and slide of the belt should be reduced to a minimum. However, the elastic extensions of conveyor belts vary according to type.

The elastic modulus of the conveyor belt is the ratio between the stress and strain of the belt; it is an important index for assessing the capability of the belt to resist elastic deformation. A higher elastic modulus often indicates that a greater stress is required to cause elastic deformation of a belt and that the rigidity of the belt will be greater. For a certain type of conveyor belt, the elastic modulus remains constant [20]. To date, numerous studies on conveyor belts have been reported. These studies have mainly focused on the resistance of a belt while running. The resistance between the belt and the rollers accounts for approximately 60% of the total resistance during belt operation [5, 7, 14, 15]. The elastic modulus of the bottom cover of the belt is also an important factor for running resistance. Given the same specifications of the conveyor belt, an increased rigidity of the bottom cover of the belt, i.e., the adoption of the bottom cover with a higher elastic modulus, can effectively reduce the indentation area of the supporting roller on the belt, thereby reducing the running resistance [21–23].

Multi-roller driving serves as an extension of single-roller driving, which is, theoretically, a result of the increasing surrounding angle between the belt and the roller. Multi-roller driving arrangements can take multiple forms, and the basic forms include double-head roller driving and single-head and tail roller driving. With the increase in the conveying distance and conveyed volume of conveyor belts, central line friction driving and combinations of the two basic arrangements have been developed to satisfy the requirements for large traction forces [19]. Due to its balanced power allocation, multi-roller driving effectively reduces the motor power of the main output under the condition of uneven power allocation, which reduces the maximum tension of the conveyor belt, thereby elongating the service life of the belt. However, the manner in which the elastic modulus of the conveyor belt influences the power allocation and the extent of this influence remains unreported.

Using typical double-head driving conveyor belts as the study object, this study investigates the influence of the elastic modulus of the belt on the driving force of the rollers. For the dynamic analysis of long-distance belt conveyors, the commonly used modeling methods include the Maxwell model, the Kelvin-Voigt model, the Standard model, and the Maxwell-Kelvin model. The Maxwell model connects springs and damping in series; however, it is applicable for only the stress analysis of the belt. Although the Standard model and Maxwell-Kelvin model can satisfactorily reflect the viscoelasticity of the belt, they are disadvantageous because they require too many parameters and a complex modeling process [6, 17]. The Kelvin-Voigt model connects springs and dampers in parallel. This model reflects not only the stress of the belt but also the deformation of the belt. Furthermore, the modeling process of the Kelvin-Voigt model is simple. Therefore, to achieve the goal of this study, the Kelvin-Voigt viscoelastic model is taken as the unit model of the conveyor belt, and a dynamic model of the belt conveyor is established. However, the Kelvin-Voigt model places particular emphasis on the belt dynamics caused by belt viscoelasticity. Belt indentation is introduced into the simulation model in the form of a resistance parameter. AMEsim simulation software is used for the dynamic analysis of the discrete model of the whole machine. DIN is used to simulate the friction coefficient, and the point-by-point tension method is utilized to calculate the resistance. Loading is performed according to the variation in the starting acceleration during the start-up process when the belt is under a full load, and sine acceleration is used in this study. By measuring the strains at the belt approach point and the runaway point from the drive roller, the driving force values for individual rollers of the conveyor belts with different elastic moduli are obtained. Actual measurements are performed to obtain the power ratio between the driving rollers; then, the actual results and the simulation results are compared.

## Methods

**Fig 1:**
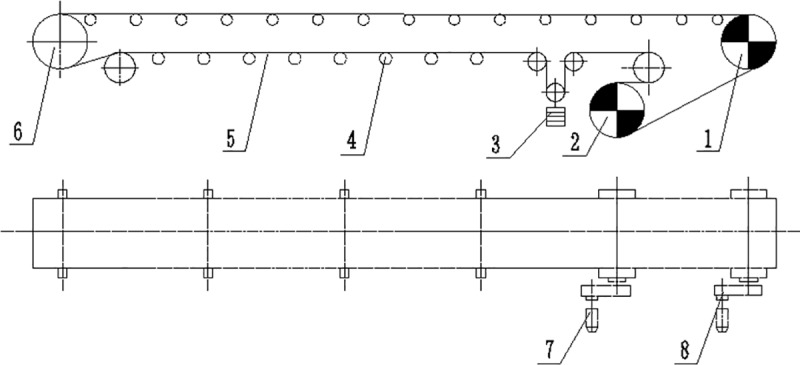
Schematic diagram of the double-head driving belt conveyor. 1: Driving roller 1. 2: Driving roller 2. 3: Tension device. 4: Supporting roller. 5: Conveyor belt. 7: Driving device 2. 8: Driving device 1.

The arrangement of the double-head rollers used in this study is shown in Fig 1.

### Dynamic model of the conveyor belt

The dynamic model of the conveyor belt was established based on the following assumptions [13]:

1. Both the rollers and supporting rollers are rigid.
2. The supporting rollers are evenly distributed, and the bulk materials on the belt are in a uniform distribution.
3. The transverse movement of the belt is ignored.

**Fig 2:**
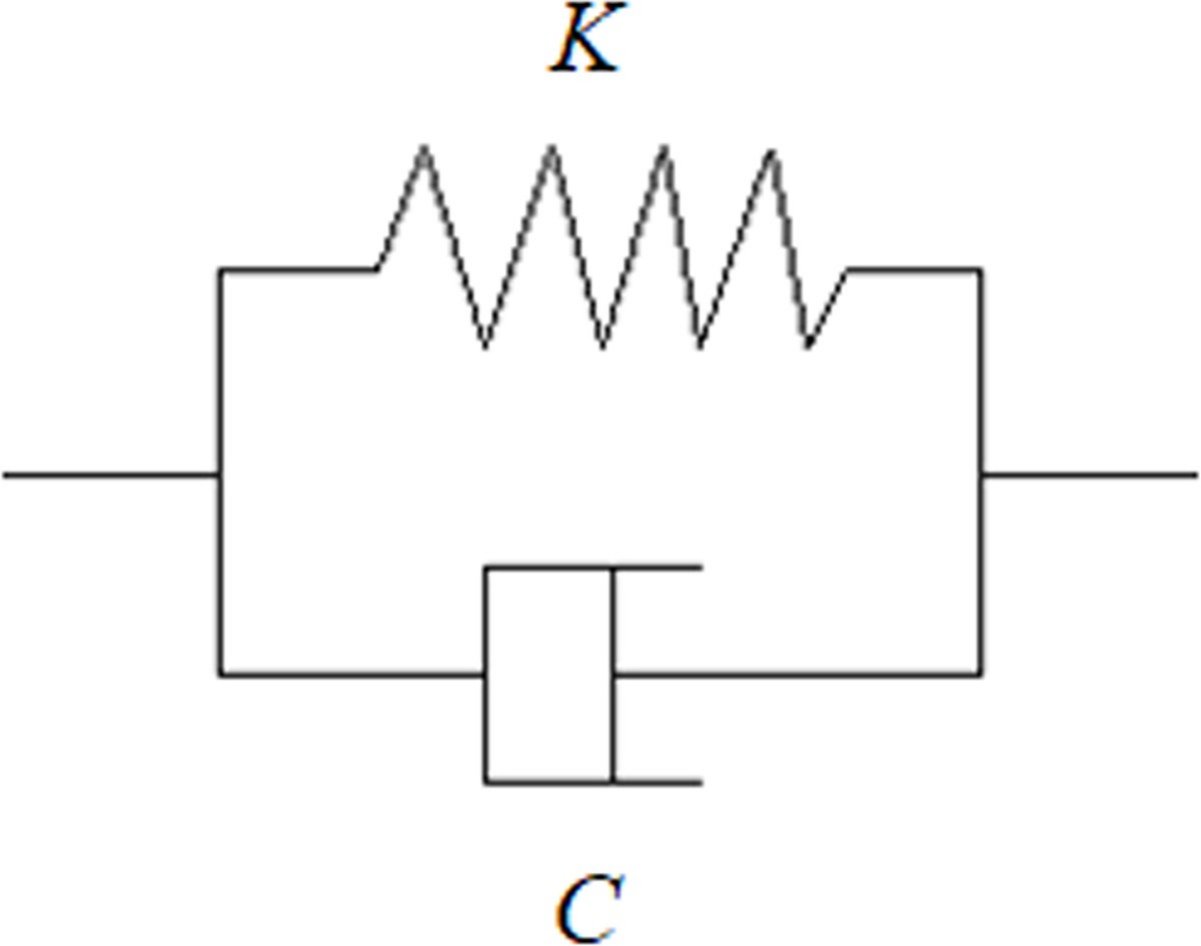
Schematic diagram of the Kelvin-Voigt model.

The Kelvin-Voigt viscoelastic model is used as the unit model of the conveyor belt, which is composed of a mass and spring damping system (Fig 2). The dynamic expression of the model is as follows [13]:

$$\sigma =\frac{E_{i}\times B}{L_{i}}\varepsilon +\frac{E_{i}\times B}{L_{i}}\tau \varepsilon$$ (1)

where *E*_*i*_ is the equivalent elastic modulus of unit *i* (N/mm), *B* is the width of the belt (mm), *L*_*i*_ is the selected unit length of the conveyor (mm), and *t* is the rheological constant (normally taken as 0.8–1).

### Elastic modulus of the conveyor belt

The belt is composed of a cover compound and the core layer. This composition determines the strong viscoelasticity of the belt. Conveyor belts with different core layers have different elastic moduli. During the start-up process of the conveyor, the elastic extension of the conveyor belt leads to an inequality between the peripheral velocities of the two head driving rollers *v*_*1*_ and *v*_*2*_, which in turn affects the driving power allocations of the two rollers, causing unequal tensions on the belt. The imbalanced power allocations may even result in driving motor burnout or early fatigue damage to the belt. Table 1 summarizes the technical parameters of the conveyor belt used in this study.

**Table 1:** Technical parameters of different types of conveyor belts [20].

Type	Elastic modulus (N/mm)	Viscoelastic dashpot (N.s/mm)
Nylon 600–3	3878.82	715.10
ST-1000	4038.81	7615.35
Canvas 650–4	4832.35	1167.04
Dacron 1067–4	8188.41	1157.65
PVG-2000S	16000.00	10377.53
ST-2000	18285.70	14497.54

### Dynamic model of the primary parts

A belt conveyor is a complex mechatronic system that is composed of traction, support, driving, tension, redirection and safety protection devices. In this study, the dynamic equations of the parts of the belt conveyor were established; then, they were incorporated into the dynamic equation of the whole system. To simplify the model, only the traction, driving and tension devices were taken into consideration during modeling [10] in this study.

#### Dynamic modeling of the conveyor belt

**Fig 3:**
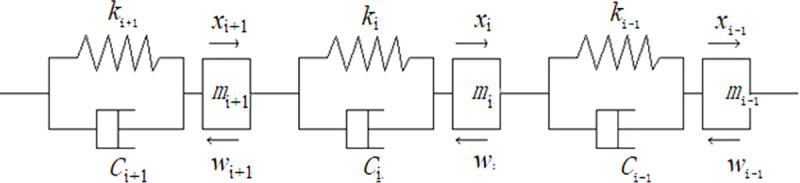
The discrete unit model of the conveyor belt.

Currently, the commonly used modeling methods for belt conveyors include Newtonian analysis, wave analysis and discretization [24]. Newtonian analysis is primarily based on the theory of rigid bodies, but conveyor belts are viscoelastic bodies. According to the Newton method, the strains of the belt along the horizontal and vertical directions should be assumed to be 0 to solve the equations of the model. However, this method neglects the indentation resistance of the belt. In contrast, wave analysis requires the consideration of the strains of the belt along the horizontal and vertical directions, as well as the indentation of the belt, to solve the equations of the model. It also requires the consideration of the propagation of wave reflections and refractions. The discretization method has an advantages over the former two methods in that it considers only the vertical stress-strain relation of the belt, ignoring the horizontal stress-strain relation [24, 25]. Furthermore, the former two methods require numerous boundary conditions to be defined, such as the displacement relationship of the driving roller between the approach point and the runaway point of the conveyor belt, the balance of the torque on the driving roller, the energy loss caused by belt deformation, the distribution of the tension along the belt, and the relative slide between the conveyor belt and the supporting roller, which makes it difficult to solve the equations of the model. Therefore, in this study, the Kelvin-Voigt viscoelastic model is adopted as the unit model, based on which finite element discretization modeling is performed. As shown in Fig 3, the established discrete unit model can be easily used to exhibit the actual situation of the conveyor belt at work.

The conveyor belt is an endless component. The discrete units shown in Fig 3 are taken as the subjects in this study. Suppose that the mass of a discrete unit is *m*_*i*_, the rigidity is *k*_*i*_, the damping coefficient is *c*_*i*_, the running resistance is *w*_*i*_, and the displacement is *x*_*i*_. The mass *m*_*i*_ contains the masses of four parts: the head, the carry segment, the return segment and the tail, which include the masses of the conveyor belt, materials, rotation parts of the supporting rollers, head driving roller and tail driving roller, with the dynamic models represented as follows [26, 27]:

1. Equivalent mass of the head:

$$m_{M+N}=\left(q_{B}+q_{G}+q_{r}\right)\times l_{M+N}+q_{1}$$ (2)

1. Equivalent mass of the carry segment:

$$m_{i}=\left(q_{B}+q_{G}+q_{r}\right)\times l_{i},(i=1,\cdots ,M-1)$$ (3)

1. Equivalent mass of the return segment:

$$m_{i}=\left(q_{B}+q_{r}^{\prime }\right)\times l_{i},\left(i=M+1,\cdots ,M+N-1\right)$$ (4)

1. Equivalent mass of the tail part:
  $$m_{M}=\left(q_{B}+q_{G}+q_{r}\right)\times l_{M}+q_{2}$$ (5)
  where *M* is the number of discrete units of the carry side of the belt, *N* is the number of discrete units of the return side, *q*_*B*_ is the mass of the unit length of the belt, *q*_*G*_ is the mass of the unit length of the bulk materials, *q*_*r*_ is the mass of the rotation part of the supporting roller at a unit length on the carry side, *q’*_*r*_ is the mass of the rotation part of the supporting roller at a unit length on the return side, *q*_*1*_ is the mass of the head driving rollers, and *q*_*2*_ is the mass of the tail driving rollers.

The Lagrangian motion differential equation of the entire conveyor belt is established according to the dynamic principles as follows [15]:

$$m_{i}{\ddot{x}}_{i}+\left(c_{i}+c_{i+1}\right){\dot{x}}_{i}-c_{i}{\dot{x}}_{i+1}-c_{i-1}{\dot{x}}_{i-1}+\left(k_{i}+k_{i-1}\right)x_{i}-k_{i}x_{i+1}-k_{i}x_{i-1}=w_{i}$$ (6)

Eq (6) is then transformed into a matrix to obtain the motion differential equation of the whole system, as follows [28]:

$$M\ddot{X}+C\dot{X}+KX=W$$ (7)

$$with\;the\;mass\;matrix\;M=\left[\begin{array}{ccccccc} m_{1} & & & & & & \\ & \ddots & & & & & \\ & & m_{i-1} & & & & \\ & & & m_{i} & & & \\ & & & & m_{i+1} & & \\ & & & & & \ddots & \\ & & & & & & m_{M+N} \end{array}\right],$$ (8)

$$the\;displacement\;matrix\;X=\left[\begin{array}{c} x_{1}\\ \vdots \\ x_{i-1}\\ x_{i}\\ x_{i+1}\\ \vdots \\ x_{M+N} \end{array}\right],$$ (9)

$$\mathrm{the}\;\mathrm{damping}\;\mathrm{matrix}\;C=\left[\begin{array}{ccccccccc} -c_{M+N} & c_{1}+c_{M+N} & -c_{2} & & & & & & \\ \cdots & \cdots & \cdots & & & & & & \\ & & -c_{i-2} & c_{i-1}+c_{i-2} & -c_{i} & & & & \\ & & & -c_{i-1} & c_{i}+c_{i-1} & -c_{i+1} & & & \\ & & & & -c_{i} & c_{i+1}+c_{i} & -c_{i+2} & & \\ & & & & & \cdots & \cdots & \cdots & \\ & & & & & & -c_{M+N-1} & c_{M+N}+c_{1} & -c_{1} \end{array}\right],$$ (10)

$$\mathrm{and}\;\mathrm{the}\;\mathrm{rigidity}\;\mathrm{matrix}\;K=\left[\begin{array}{ccccccccc} -k_{M+N} & k_{1}+k_{M+N} & -k_{2} & & & & & & \\ \cdots & \cdots & \cdots & & & & & & \\ & & -k_{i-2} & k_{i-1}+k_{i-2} & -k_{i} & & & & \\ & & & -k_{i-1} & k_{i}+k_{i-1} & -k_{i+1} & & & \\ & & & & -k_{i} & k_{i+1}+k_{i} & -k_{i+2} & & \\ & & & & & \cdots & \cdots & \cdots & \\ & & & & & & -k_{M+N-1} & k_{M+N}+k_{1} & -k_{1} \end{array}\right]$$ (11)

#### Dynamic models of the driving and tension devices

**Fig 4:**
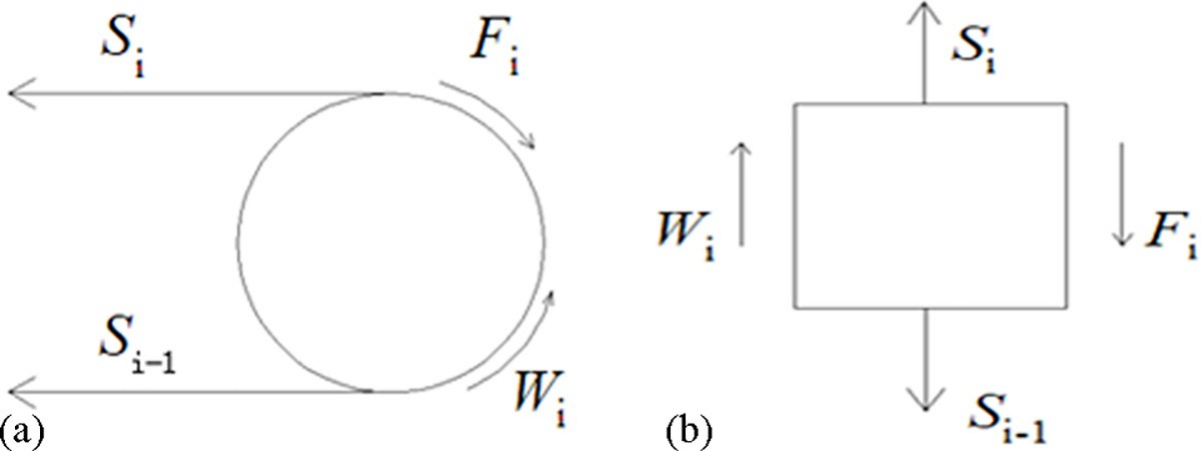
The dynamic model of the driving roller. (a) Force analysis of the driving roller. (b) Simplified model of the driving roller.

The driving device, as the power source of the whole conveyor belt, experiences the actions of multiple forces. Driving rollers make a circular motion, and the established model is shown in Fig 4.

The dynamic motion equation of the driving roller is as follows:

$$F_{i}\frac{D}{2}+S_{i-1}\frac{D}{2}-W_{i}\frac{D}{2}-S_{i}\frac{D}{2}=J\ddot{\theta }$$ (12)

where *F*_*i*_ is the driving force of the ith driving roller, *S*_*i*_ and *S*_*i-1*_ are the tensions of the two sides of the roller, *W*_*i*_ is the resistance of the roller, and *D* is the diameter of the roller.

**Fig 5:**
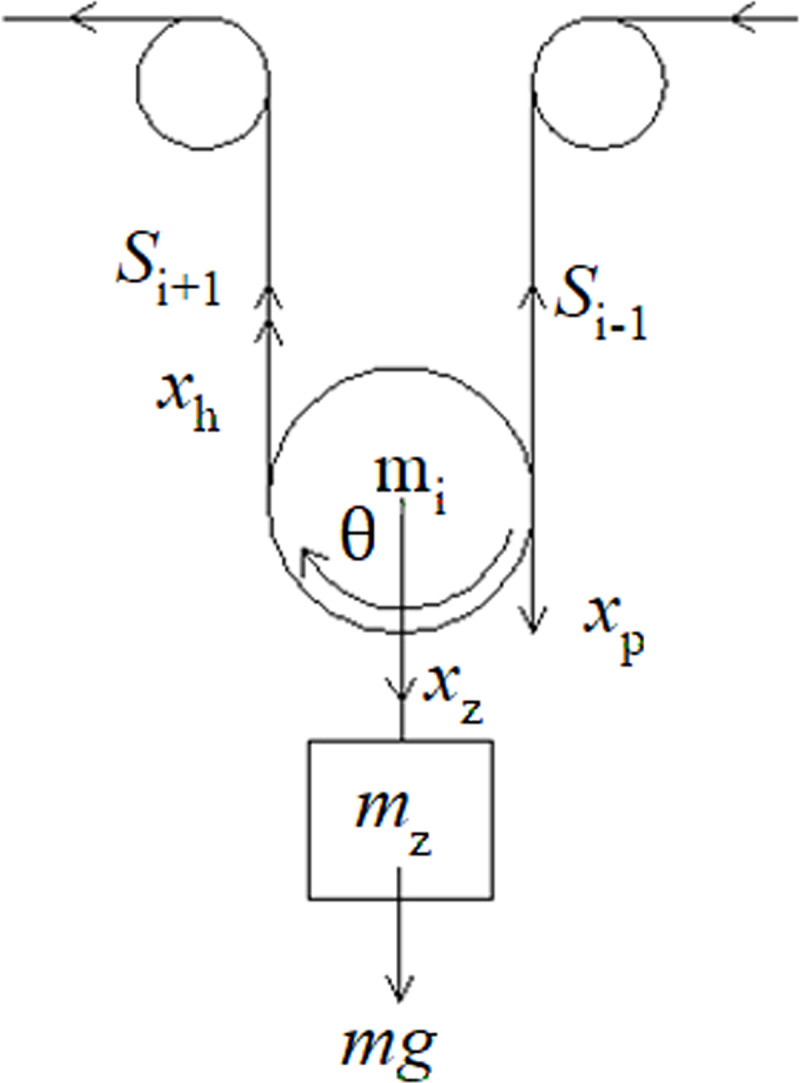
Stress analysis of the tension device.

The selection of the tension device should be based on the space and the route arranged for the conveyor. Gravity take-up units are used in this study. Conveyor belts rely on the friction between the bottom covering rubber surface and the driving rollers, and the tension device provides a contact force for the conveyor and the rollers, which enables the conveyor belt to obtain a large friction force and avoid slippage with the rollers. The tension device affects the performance of the conveyor belt, particularly the tension, which directly affects the selection of the conveyor belt type [12]. Therefore, reasonable modeling has a determinative effect on the simulation effect of the conveyor. Fig 5 shows the actual loading on the gravity take-up units.

Suppose that there is no slippage between the tensioner rollers and the conveyor belt. Based on Fig 5, the stress on a tensioner roller can be expressed as follows:

$$m_{i}{\ddot{x}}_{z}=m_{z}g-\left(s_{i-1}+s_{i+1}\right)$$ (13)

$$J\ddot{\theta }=s_{i+1}r-s_{i-1}r+F_{r}r$$ (14)

where *m*_*i*_ = *m*_*c*_ + *m*_*z*_, where *m*_*z*_ is the mass of the gravity take-up and *m*_*c*_ is the mass of the tensioner roller, xz represents the displacement of the tensioner roller, *J* represents the moment of inertia of the tensioner roller, *r* is the radius of the tensioner roller, *Fr* is the resistance on the shaft of the tensioner roller, and θ is the angular displacement of the tensioner roller.

**Fig 6:**
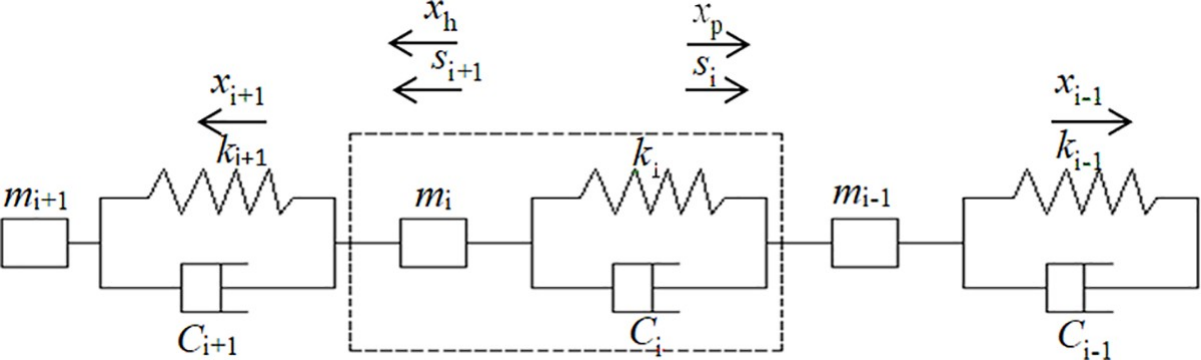
The discrete dynamic model of the tensioner device of the conveyor belt.

Because conveyor belts are viscoelastic, the gravity take-up will oscillate up and down according to the changes in the tension of the conveyor belt under conditions such as conveyor belt start-up, belt braking and a sudden change in the load on the conveyor belt. Due to the difference in the strain of the conveyor belt at the approach point and the runaway point of the tensioner roller, the speed of the belt varies. Therefore, the speeds at the approach point and runaway point of the tensioner roller are not the same. Based on these characteristics, the discrete dynamic model of the tensioner device is established [29] (Fig 6).

If the displacements at the approach point and runaway point of the discrete unit of the tensioner roller *i* are *x*_*p*_ and *x*_*h*_, respectively, then the tensions *s*_*i*_ and *s*_*i+1*_ can be expressed as follows:

$$s_{i}=k_{i}\left(x_{i}-x_{p}\right)+c_{i}\left({\dot{x}}_{i}-{\dot{x}}_{p}\right)$$ (15)

$$s_{i+1}=k_{i+1}\left(x_{h}-x_{i+1}\right)+c_{i+1}\left({\dot{x}}_{h}-{\dot{x}}_{i+1}\right)$$ (16)

According to rotation of the roller, the following can be obtained:

$$x_{h}=\theta (t)r+m_{z}$$ (17)

$$x_{p}=\theta (t)r-m_{z}$$ (18)

Then, the following can be obtained:

$$m_{z}{\ddot{x}}_{z}=m_{z}g-k_{i}x_{i}+k_{i}x_{p}-k_{i+1}x_{h}+k_{i+1}x_{i+1}-c_{i}{\dot{x}}_{i}+c_{i}{\dot{x}}_{p}-c_{i+1}{\dot{x}}_{h}+c_{i+1}{\dot{x}}_{i+1}$$ (19)

$$J\ddot{\theta }=\left(K_{i}x_{i}-k_{i}x_{p}-k_{i+1}x_{h}+k_{i+1}x_{i+1}\right)+\left(c_{i}{\dot{x}}_{i}-c_{i}{\dot{x}}_{p}-c_{i+1}{\dot{x}}_{h}+c_{i+1}{\dot{x}}_{i+1}\right)r+F_{T}r$$ (20)

The displacement relations among the approach point, the runaway point and the gravity take-up are as follows:

$$x_{p}{=x}_{h}{-2m}_{z}$$ (21)

$$x_{h}{=x}_{p}{+2m}_{z}$$ (22)

Then,

$$m_{z}x_{z}+k_{i}x_{i}-k_{k}x_{p}+k_{i+1}x_{h}-k_{i+1}x_{i+1}+c_{i}{\dot{x}}_{i}-c_{i}{\dot{x}}_{p}+c_{i+1}{\dot{x}}_{h}-c_{i+1}x_{i+1}=m_{z}g$$ (23)

$$m_{G}{\ddot{x}}_{p}-k_{i}x_{i}+\left(k_{i}+k_{i+1}\right)x_{p}+2k_{i+1}x_{z}-k_{i+1}x_{i+1}-c_{i}{\dot{x}}_{i}+\left(c_{i}+c_{i+1}\right){\dot{x}}_{p}+2c_{j+1}{\dot{x}}_{z}-c_{i+1}{\dot{x}}_{i+1}+m_{C}{\dot{x}}_{z}=F_{T}$$ (24)

$$m_{G}{\ddot{x}}_{h}-k_{i}x_{i}+\left(k_{i}+k_{i+1}\right)x_{p}-2k_{i}x_{z}-k_{i+1}x_{i+1}-c_{i}{\dot{x}}_{i}+\left(c_{i}+c_{i+1}\right){\dot{x}}_{h}+2c_{i}{\dot{x}}_{z}-c_{i+1}{\dot{x}}_{i+1}-m_{G}{\dot{x}}_{z}=F_{T}$$ (25)

### The simulation model and its parameters

AMEsim simulation software was utilized to dynamically analyze the discrete model of the entire belt conveyor. The parameters were set based on the primary parameters of mining belt conveyors, and the Harrison starting speed was used as the input for the conveyor, which has a minimum mechanical impact [26]. The conveyor was equipped with different types of conveyor belts; then, the obtained images were investigated to determine the influence of the viscoelasticity of the conveyor belt on the power equilibrium of multiple drives.

#### The discrete model of the whole machine

**Fig 7:**
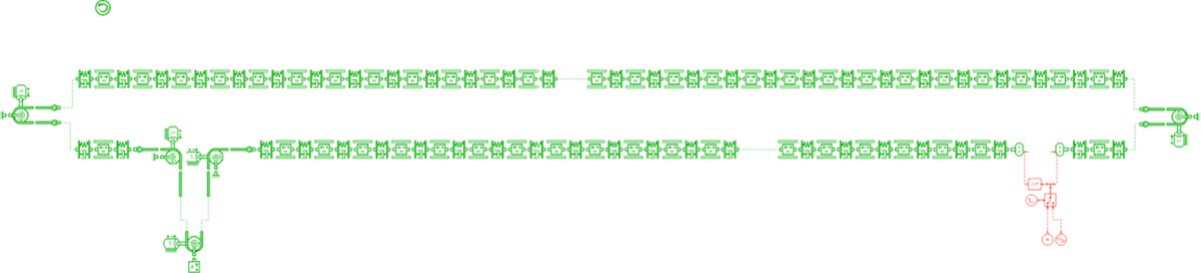
Diagram of the AMEsim discrete model of the belt conveyor.

The whole conveyor system was equally partitioned into a finite number of head-tail-connected closed-circuit subsystems with AMEsim. Because the tension curve and the gravity take-up curve converge when the unit length is less than 13 m [26], an interval of 13 m was used for unit partitioning to establish the conveyor model, which is shown in Fig 7.

#### Parameter setting for the simulation

The long-distance multi-drive belt conveyor at a mine was taken as an example. The main technical parameters are summarized in Table 2.

**Table 2:** The main parameters of the conveyor.

Item	Value	Item	Value
Conveyor length	1700 m	Driving form	Double-head roller
Convey dip angle	0°	Power ratio	1:1
Convey volume	1500 t/h	Equivalent mass of the carry-side supporting roller	26.13 kg/m
Speed	3 m/s	Equivalent mass of the return-side supporting roller	18.75 kg/m
Conveyor belt width	1400 mm	Mass of unit length of the bulk materials	180.15 kg/m
Simulated frictional resistance coefficient	0.022	Rheological constant of the conveyor belt	0.80
Conveyor belt mass	42 kg/m	Start-up time of the conveyor belt [19, 20]	90 s

Conveyor belts with different elastic moduli were selected. For the sake of a convenient calculation and simulation analysis, the mass of the working conveyor belts under a full load was set to 42 kg/m. Additionally, the Harrison starting speed curve was used as the input for the conveyor. The acceleration and speed were calculated as follows [14]:

$$a(t)=\frac{v\pi }{2T}\sin\frac{\pi t}{T}(0\leq t\leq T)$$ (26)

$$v(t)=\frac{v}{2}\left(1-\cos\frac{\pi t}{T}\right)(0\leq t\leq T)$$ (27)

### Actual measurements

The simulation results were compared with actual measurements.

**Fig 8:**
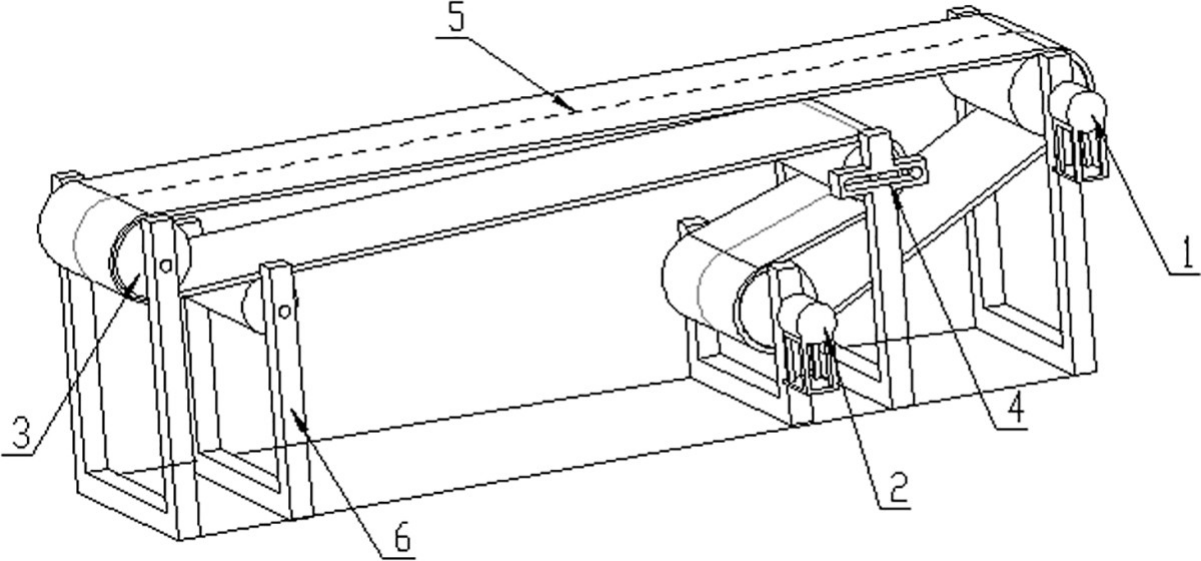
Experimental schematic diagram of the double-head driving belt conveyor. 1: Inverter fed motor 1. 2: Inverter fed motor 2. 3: Tail redirection roller. 4: Tensioner. 5: Conveyor belt. 6: Stand.

**Fig 9:**
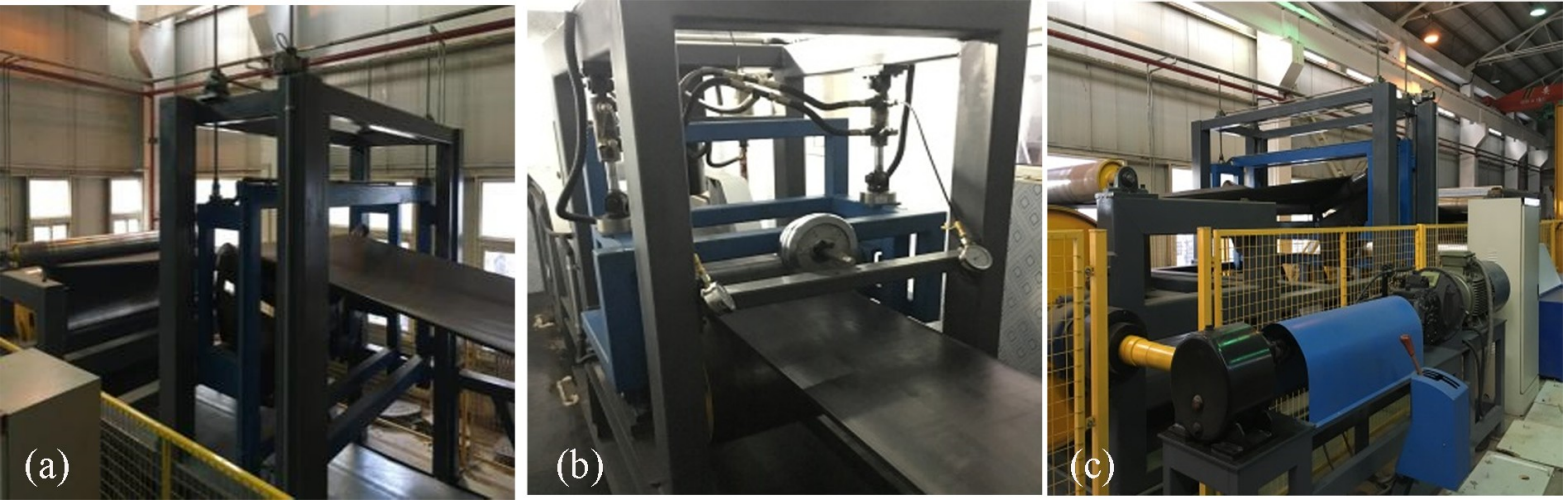
Experimental devices of the double-head driving belt conveyor.

The experimental principle of the double-head driving belt conveyor is illustrated in Fig 8, and the experimental devices are shown in Fig 9. All the rollers and stands of the experimental system were easy to disassemble and assemble, satisfying the requirement for fast conveyor replacement. A torque transducer was installed at one end of the driving roller shaft, which was used to measure the torque of the driving roller *M*. Based on the obtained *M* value, the driving force of the roller *F* was obtained according to the following equation:

$$M=F\frac{D}{2}$$ (28)

## Results and discussion

Three-dimensional models were established based on the elastic moduli of the conveyor belts with different cores (canvas, Dacron and wire rope). Then, computer simulations were performed to analyze the surge phenomenon of the conveyor belts at the time of conveyor start-up. The drive sharing of individual driving rollers, as well as the specific ratio between the power transmitted by the two rollers, was obtained.

### Simulation results

**Fig 10:**
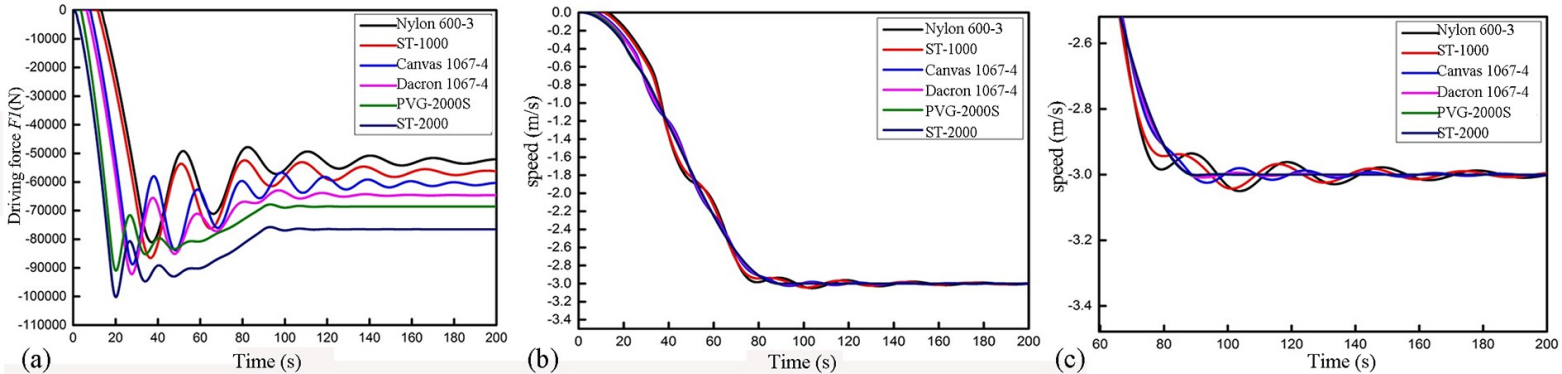
The relation of the transmitted driving force and the speed of the first roller with time. (a) The driving forces of the first roller for the conveyor belts with different elastic moduli. (b) The results of the time required for stabilization of the first roller for the conveyor belts with different elastic moduli. (c) Magnification of panel (b).

**Fig 11:**
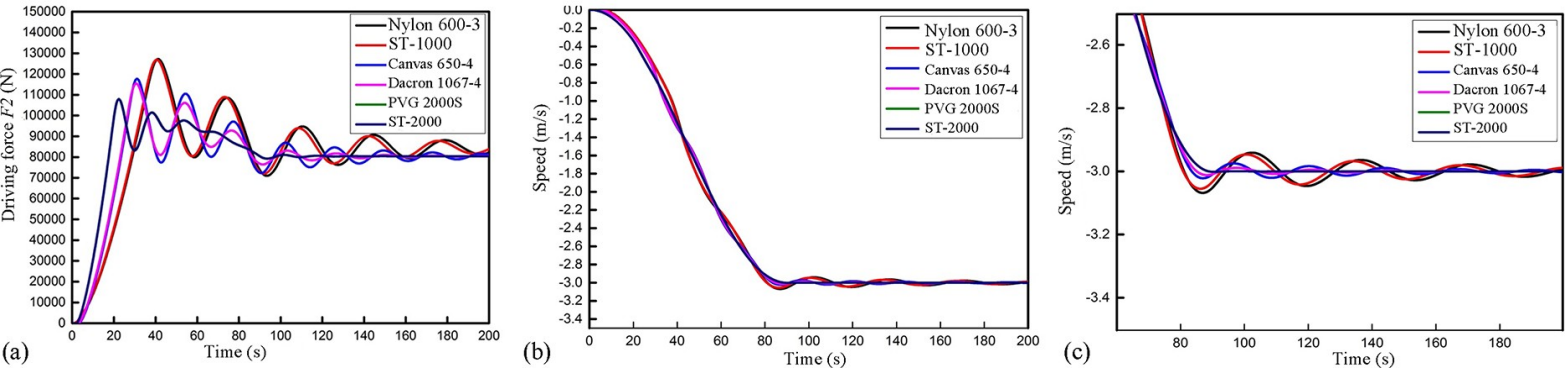
The relation of the transmitted driving force and the speed of the second roller with time. (a) The driving forces of the second roller for the conveyor belts with different elastic moduli. (b) The results of the time required for stabilization of the second roller for the conveyor belts with different elastic moduli. (c) Magnification of panel (b).

This study focused on the influence of conveyor belts with different elastic moduli under full loads on the drive sharing of double rollers at the time of start-up. The speeds and drive sharing of the rollers corresponding to the conveyor belts with different elastic moduli are shown in Figs 10 and 11.

Different acceleration times have different impact forces on the conveyor belts under full loads during start-up. The longer the start-up time is, the smaller the impact upon the conveyor belt will be. However, in practice, the start-up time is not allowed to continue indefinitely. Therefore, the start-up time for the conveyor belt was set to 90 s in this study.

As shown in Figs 10 and 11, for conveyor belts with different elastic moduli, the drive sharing and the drive force stabilizing times of the two rollers were noticeably different when the conveyor belt was under a full load. Fig 10 shows the transmitted drive force and drive force stabilizing time of the first roller for different conveyor belts. The nylon belt showed the most apparent fluctuations among the belts tested, with a maximum tension of 86,088.83 N. The belt began to show gradual tension after 13.27 s of electrification, and the time required for belt stabilization was approximately 220 s. In contrast, although the maximum tension of the ST-2000 steel wire belt was larger (the peak value reached 98,588.44 N), tension began after 5.17 s of electrification, and the time required for belt stabilization was no longer than 95 s. Therefore, under a full load, the drive force shared by the first roller *F*_*1*_ increased with the increase in the elastic modulus of the conveyor belt. Fig 10A shows a noticeable delay between the electrification process and when the force starting to increase. The reason underlying this phenomenon may be as follows. When the motor starts the roller, the belt experiences a sudden force after being in a static state. As the belt possesses viscoelastic properties, the belt will extend elastically within a short period, which leads to relative slippage between the belt and the roller. The belt can then run stably when it is fully tensioned, and the static friction force experienced by the belt reaches a maximum. Since the belts with different elastic moduli have different extensions, the time when the static friction force reaches the maximum value will be different according to the elastic moduli, which is reflected by the different delays required for the circumferential forces to reach their maximum values. Fig 11 shows the transmitted drive force and drive force stabilizing time of the second roller. When the nylon core conveyor belt is used, the maximum tension under a full load is 85,120.37 N, the tension time for the second roller is 4.57 s, and the time required for conveyor stabilization is 220 s. However, when the ST-2000 steel wire core belt is used, the maximum tension reaches 112,110.81 N, the tension time is reduced to 2.1 s, and the time required for conveyor stabilization is 95 s. These results indicate that during start-up under a full load, the drive force shared by the second roller *F*_*2*_ decreases with the increase in the elastic modulus. This is because the upper and lower branch conveyor belts transmit forces towards the machine tail in a wave manner under the traction of the driving rollers. Conveyor belts with different rigidities and damping have different decreasing effects on the surge phenomenon. With the decrease in the elastic modulus, the surge phenomenon of the conveyor becomes increasingly substantial (the surge phenomenon of the conveyor belt has a nearly positive correlation with the elastic modulus); therefore, the time required for the conveyor start-up to stabilize is long.

### Experimental results

The experimental results are summarized in Table 3.

**Table 3:** The power ratio of the conveyor belts with different elastic moduli according to the simulation results and the actual measurements.

Conveyor belt type	Elastic rigidity *k* (N/m)	Speed of roller 1 *v*_1_ (m/s)	Driving force of roller 1 *F*_1_ (N)		Speed of roller 2 *v*_2_ (m/s)	Driving force of roller 2 *F*_2_ (N)		Actual power ratio *P*_1_/*P*_2_	
			Simulation	Actual measurement		Simulation	Actual measurement	Simulation	Actual measurement
Nylon 600–3	417,719	3	52,946.71	53,249.98	3	85,120.37	92,122.35	1.60	1.73
ST 1000	434,949	3	56,491.83	57,005,16	3	83,702.92	87,787.81	1.48	1.54
Canvas 650–4	520,406	3	60,962.11	63,593.32	3	81,010.67	89,030.64	1.32	1.40
Dacron 1067–4	881,829	3	64,586.36	66,001.41	3	80,545.44	87,781.84	1.24	1.33
PVG-2000S	1,723,076	3	68,582.92	70,012.44	3	80,414.52	85,415.11	1.17	1.22
ST 2000	1,969,229	3	76,448.25	77,853.93	3	80,214.31	84,860.73	1.05	1.09

As shown in Table 3, the values obtained by the simulation were slightly lower than the actual measurements. The reason for this inconsistency is that the influences of some factors were ignored during the theoretical calculation, and all the parameters were given constant values. However, in practice, an uneven distribution of the mass of the rotation parts of the supporting rollers may exist due to the factors related to manufacturing, installation and operation. In addition, material loading may also lead to a deviation between the linear load of unit length and the set value. Nevertheless, the theoretical calculation and the actual measurements exhibited changing trends that were basically consistent. For the nylon, ST-1000 and canvas conveyor belts, the driving force of the second roller after stabilization *F*_*2*_ was considerably greater than the driving force of the first roller after stabilization *F*_*1*_, with a ratio of 1.60 according to the simulation results and a ratio of 1.73 according to the actual measurements.

With the increase in the elastic modulus of the belt, the ratio between the driving force shared by the first roller and that shared by the second roller gradually decreased. Specifically, the ratios according to the simulation results and the actual measurements were 1.24 and 1.33 for the Dacron conveyor belt, 1.17 and 1.22 for PVG-2000S, and 1.05 and 1.09 for ST-2000, respectively. The total power of the conveyor was determined by the friction force principle, which satisfied Euler’s formula. Therefore, with the given wrap angle of the drive pulleys and friction coefficient, the total power of the conveyor remained unchanged and was equivalent to the sum of the power of the multi-roller driving motors. These results indicate that during the starting process under a full load, with the increase in the elastic modulus, the ratio between *F*_1_ and *F*_2_ in the double-head drive gradually approached the theoretical allocation ratio.

Based on the AMEsim simulation experiment, the selection of a conveyor belt with a reasonably high elastic modulus for multiple drives can effectively improve the stability of the conveyor after start-up under a full load. Furthermore, with the increase in the elastic modulus, the driving sharing of the rollers approaches a balanced state. In a study conducted by Zhao et al. [7], a steel wire core conveyor belt with elongation percentages of 0, 0.01, 0.015 and 0.02 and a nylon core conveyor belt with elongation percentages of 0, 0.02, 0.03 and 0.04 were utilized to investigate the imbalanced drive sharing of a double-head driving belt conveyor, and these researchers found that the imbalanced drive sharing increased as the elongation percentage increased. The results of this study were consistent with their findings, demonstrating the feasibility of the method used in this study.

## Conclusion

This study led to the following conclusions:

1. For double-head driving belt conveyors, the elastic modulus of the belt has an important influence on the start-up process of the conveyor under a full load. As the elastic modulus increases, the rigidity of the conveyor belt increases, and the severity of the surge phenomenon during conveyor start-up is reduced.
2. The elastic modulus of the conveyor belt has a substantial influence on the power allocation of multiple drives. With the increase in the elastic modulus, the drive sharing of the double-roller drive approaches an ideal power allocation.
3. With multiple drives, the fabric core conveyor belt has a greater influence on the power allocation of the driving rollers than that of the steel wire core conveyor belt. In practice, appropriate steel wire core conveyor belts can be used to achieve reasonable power allocation.

The key to the design of belt conveyors lies in the selected type of conveyor belt, which is the basis for the selection of other parts. Based on the results of this study, a conveyor belt with a low elastic modulus is recommended in conveyor design to reduce the influence of the fluctuation in and extension of the conveyor belt on the drive sharing of the rollers during belt braking. This selection not only increases the service life of the conveyor belt but also prevents the motor from burning out due to imbalanced drive sharing, thereby realizing light weight and green conveying.
